# Beyond rhythm disorder: a rare case report of a young patient with mediastinal bronchogenic cyst

**DOI:** 10.3389/fcvm.2026.1746006

**Published:** 2026-03-06

**Authors:** Monika Shumkova, Kristina Stoyanova, Ivan Andreev, Raia Ivanova, Kiril Karamfiloff

**Affiliations:** 1 Faculty of Medicine, Internal Medicine, Medical University, Sofia, Bulgaria; 2 Department of Cardiology, UMHAT Alexandrovska Hospital, Sofia, Bulgaria

**Keywords:** bronchogenic cyst, atrial fibrillation, echocardiography, cardiac magnetic resonance imaging, congenital heart disease

## Abstract

**Background:**

Mediastinal bronchogenic cysts are sporadic congenital tumors, accounting for approximately 10%–15% of all mediastinal masses. In most cases, they remain asymptomatic. We present a case of a young man with new-onset atrial fibrillation caused by extra cardiac compression of the left atrium by mediastinal bronchogenic cyst. We report the first documented case in our country of a patient with a mediastinal bronchogenic cyst presenting with cardiac symptoms.

**Case summary:**

A 39-year-old man was admitted to the emergency department with palpitations, dyspnea, and tachycardia that developed after physical activity. Electrocardiography demonstrated the first episode of atrial fibrillation. Echocardiography revealed an oval echolucent mass compressing the left atrium. Cardiac magnetic resonance imaging showed a massive mediastinal bronchogenic cyst causing compression of the left atrium, right inferior pulmonary vein, and esophagus, which was displaced to the left side. Robot-assisted thoracoscopic surgery was planned and performed successfully.

**Discussion:**

Although typically asymptomatic, bronchogenic cysts can acutely present with life-threatening complications, particularly when they compress important cardiovascular structures. This case highlights the important role of comprehensive evaluation in young patients. It is crucial to consider extra cardiac causes in new-onset atrial fibrillation, especially in young adults. Fast and accurate diagnosis is essential to prevent further complications, with management extending beyond restoring sinus rhythm.

## 1 Introduction

Mediastinal bronchogenic cysts are sporadic congenital tumors, accounting for approximately 10%–15% of all mediastinal masses. They arise during embryogenesis because of abnormal budding of the ventral diverticulum of the foregut or the tracheobronchial tree. In most cases, they remain asymptomatic (1). In this case, the primary presentation was an acute rhythm disorder caused by the compression of the left atrium (LA). To our knowledge, this is the first reported clinical case in our country of a patient with bronchogenic cyst and cardiac symptoms.

## 2 Patient presentation

A previously healthy 39-year-old man presented to the emergency department with symptoms of palpitations, fatigue, shortness of breath, and a rapid heart rate that began following physical activity. He also reported a dry cough persisting for approximately 3 weeks. Similar episodes had occurred in the past, but of shorter durations and did not require a medical appointment. He had no medical history of cardiovascular or concomitant disease and was not on any medical therapy.

**Figure 1:**
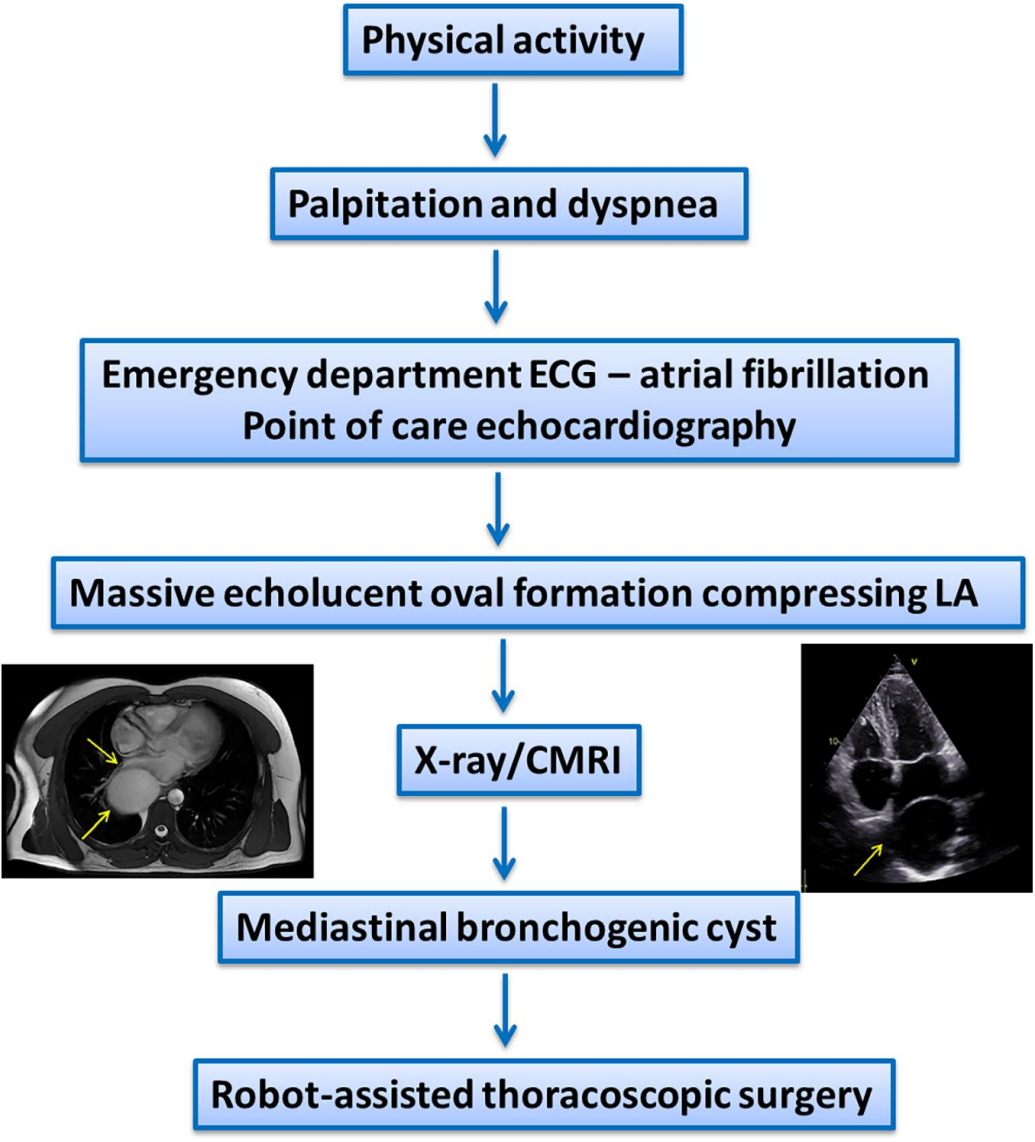
Timeline of the diagnosis.

On physical examination, the patient exhibited an irregular heart rhythm, a heart rate of approximately 100–110 bpm, slightly elevated blood pressure (140/80 mmHg), and no heart murmurs. There were no signs of central or peripheral congestion (Figure 1).

## 3 Diagnostic assessment

**Figure 2:**
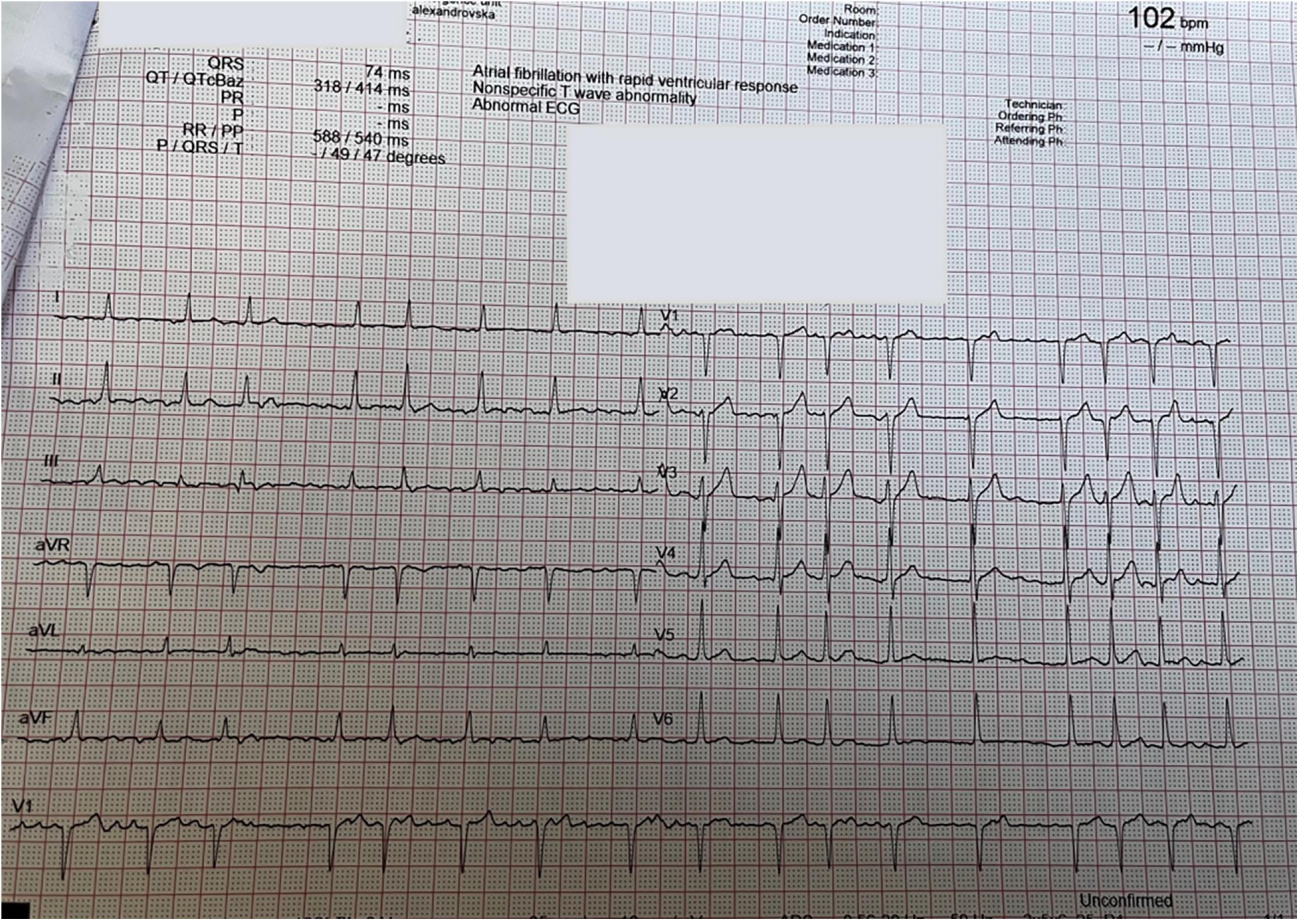
Electrocardiogram—atrial fibrillation, normal axis deviation with a ventricular rate around 110/min, and no ST-segment abnormalities.

**Figure 3:**
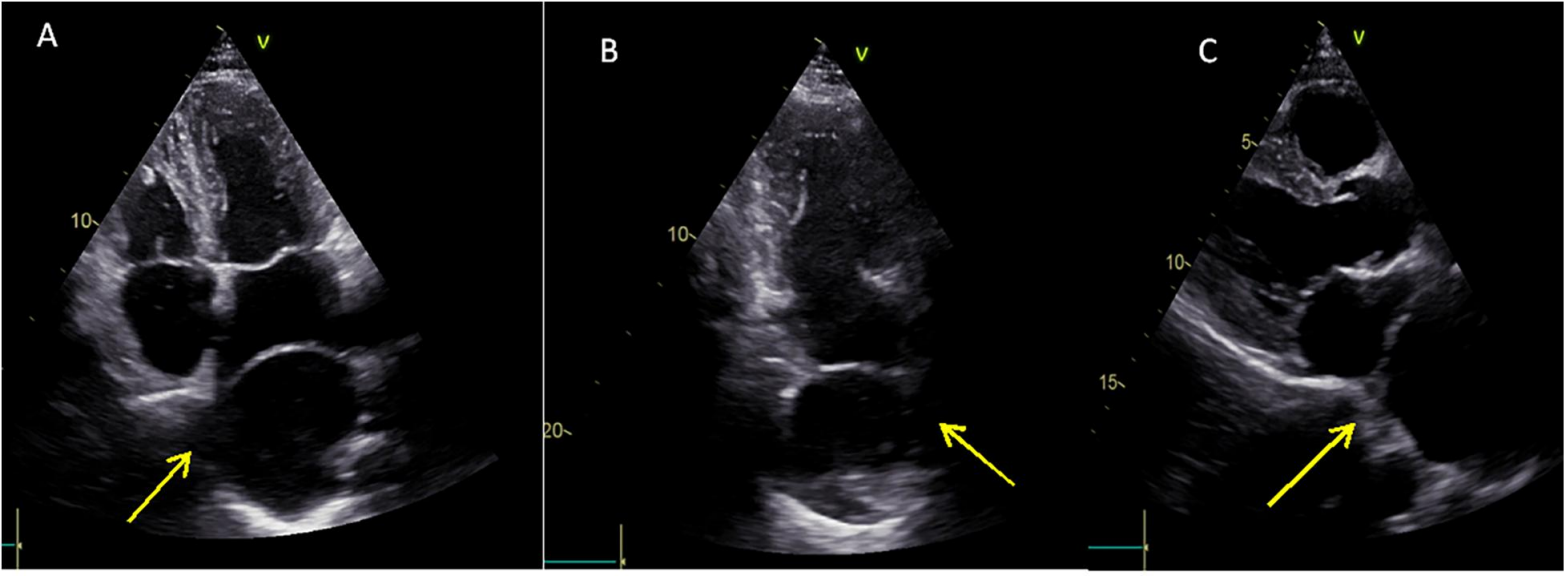
Transthoracic echocardiography—four-chamber view **(A)**, two-chamber view **(B)**, and parasternal long-axis view **(C)** showing an extra cardiac echolucent cavity that compresses the left atrium (yellow arrows).

Initial electrocardiography (ECG) revealed atrial fibrillation, normal axis deviation with a ventricular rate around 110/min, and no ST-segment abnormalities (Figure 2). Laboratory tests were unremarkable. Transthoracic echocardiography identified an echolucent extra cardiac cavity compressing the LA with no communication between the cavity and LA on color Doppler imaging (Figure 3, Supplementary Video S1).

Chest X-ray confirmed the presence of a massive oval formation in the mediastinum.

**Figure 4:**
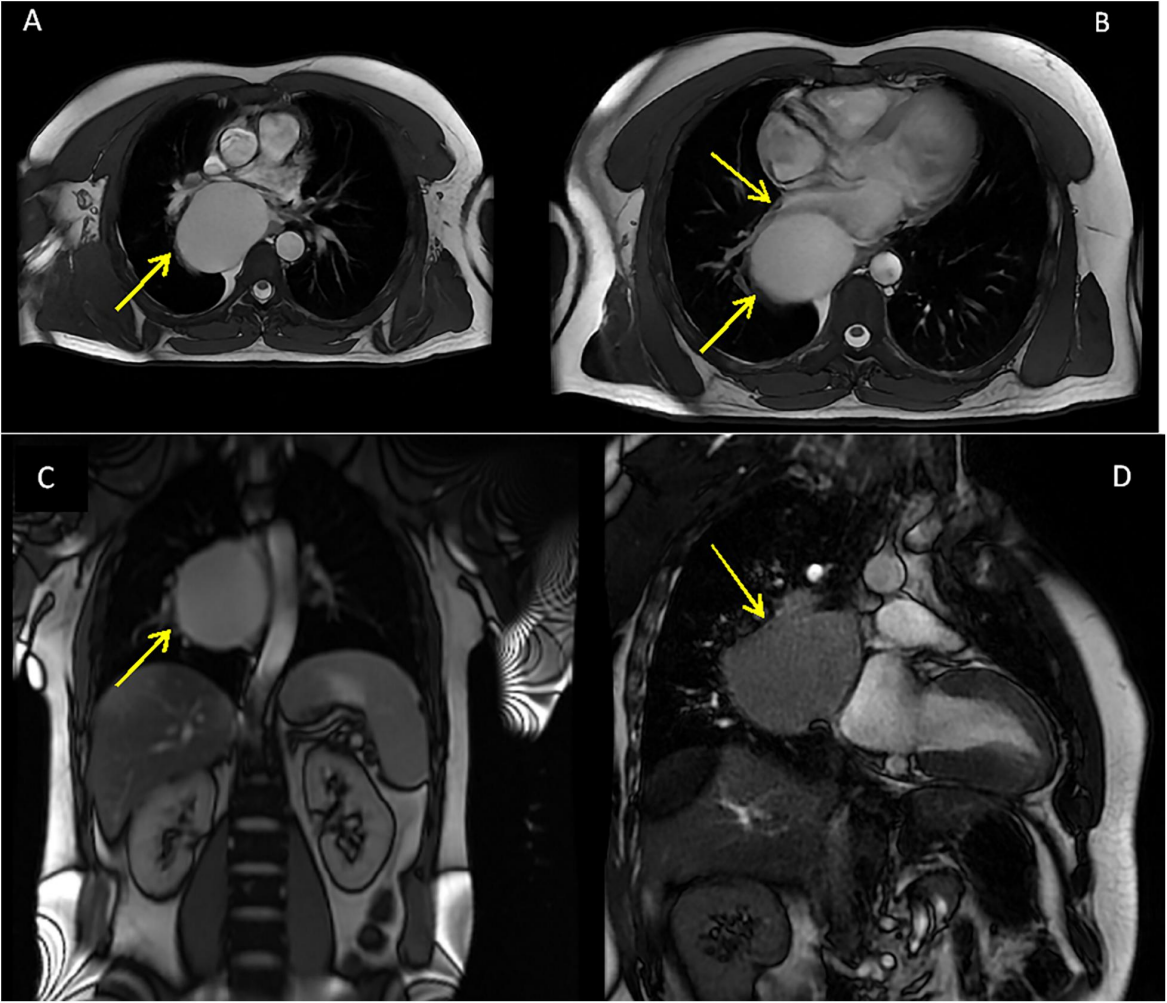
Cardiac magnetic resonance image with yellow arrows showing the oval formation: **(A)** Compression of the left inferior pulmonary vein. **(B)** Compression of left atrium and right inferior pulmonary vein. **(C)** Compression of the esophagus. **(D)** Position of the mediastinal cyst next to the left atrium.

Subsequent cardiac magnetic resonance imaging showed a massive oval formation located in the posterior mediastinum with axial diameter of 80 × 61 mm and cranio-caudal diameter of 78 mm. It was positioned on the right side of the spine, causing compression of the left atrium, right inferior pulmonary vein, and esophagus, which was displaced to the left side. No calcification or septations were observed (Figure 4).

Differential diagnosis included a lung abscess, hydatid (echinococcal) cyst, and neoplasm.

## 4 Therapeutic intervention

The patient's sinus rhythm was spontaneously restored. Anticoagulation therapy and propafenone were initiated for rhythm control. A small dose of an ACE inhibitor was started for blood pressure control. The patient was referred for elective surgical evaluation and treatment.

Severe physical exertion was stopped before the surgery. One week after the diagnosis, robot-assisted thoracoscopic (RATS) surgery was successfully performed.

In the postoperative period, the patient had one short episode of paroxysmal atrial fibrillation, resolved with intravenous propafenone. Antihypertensive therapy, antiarrhythmic therapy, and anticoagulation were continued.

## 5 Follow-up

During 1-year follow-up, the patient remained in sinus rhythm without recurrent episodes of atrial fibrillation or other cardiac symptoms.

## 6 Discussion

Congenital mediastinal bronchogenic cysts originate from the ventral foregut during embryogenesis and typically remain asymptomatic. They contain air and/or mucoid material. Approximately 70% of thoracic bronchogenic cysts are pulmonary and around 30% are mediastinal (2). Symptoms vary depending on the size of the cyst and the compression effect of structures localized around it. Most cases are discovered incidentally during X-ray, CT, or ultrasound examination performed for unrelated reasons. Common symptoms in symptomatic patients include hemoptysis, dysphagia, pneumothorax, pneumonia, hoarseness, and infection (3, 4). Acute presentations are rare. In uncommon cases, malignant degeneration of the cyst could occur (2). Infections are the most common complication.

We report a rare case of a young man with a large bronchogenic cyst with a compression of the left atrium and right inferior pulmonary vein, causing atrial fibrillation. Several similar cases have been described in the literature of patients with bronchogenic cysts and cardiac presentation. Kennebeck et al. (5) published a case report of a patient with an acute presentation with anterolateral myocardial infarction due to compression of the left main coronary artery. Han et al. (6) described a 49-year-old patient with a mediastinal cyst with compression of LA, atrial fibrillation, and a large amount of pericardial effusion. Surgical resection resulted in complete clinical recovery.

There is no consensus regarding the management of bronchogenic cysts. There are two options—surgery or conservative management with close follow-up. We advocate for early removal after diagnosis in order to prevent future complications like compression, infection, and fistulas. In this case, due to the presence of symptoms, surgical treatment was the preferred choice. The atrial fibrillation was attributed to mechanical compression. As the episodes were symptomatic, flecainide was initiated in order to keep sinus rhythm until the surgery. The antiarrhythmic drugs were continued even after the surgery.

In conclusion, bronchogenic cysts in the adult population are usually asymptomatic, but acute presentations can be life-threatening. Comprehensive evaluation of patients presenting with atrial fibrillation, even at a young age, is essential for proper diagnosis. Diagnostic tools like echocardiography can be critical in identifying underlying causes. Point-of-care echocardiography in the emergency department can rapidly make the final diagnosis and alter management, because, sometimes, restoring sinus rhythm is not the final step in the management.

## Data Availability

The raw data supporting the conclusions of this article will be made available by the authors without undue reservation.

## Supplementary material

Supplementary Video S1 Transthoracic echocardiography—apical four-chamber view shows color flow Doppler without communication between the left atrium and the extra cardiac mass.
